# A systematic review on occupational hazards, injuries and diseases among police officers worldwide: Policy implications for the South African Police Service

**DOI:** 10.1186/s12995-018-0221-x

**Published:** 2019-01-21

**Authors:** Gift Gugu Mona, Moses John Chimbari, Charles Hongoro

**Affiliations:** 1 College of Health Sciences, George Campbell Building, Mazisi Kunene Road, Howard College Campus, University of KwaZulu-Natal, P.O Box X7, Congella, Durban, KwaZulu-Natal 4013 South Africa; 2 Research Use and Impact Assessment, Human Sciences Research Council Building, 134 Pretorius Street, Pretoria, 0083 South Africa

**Keywords:** Police and occupational diseases, Occupational hazards, Occupational health and safety, Occupational injuries, Stress, Work injury

## Abstract

**Background:**

Occupational hazards, injuries and diseases are a major concern among police officers, including in Sub-Saharan Africa. However, there is limited locally relevant literature for guiding policy for the South African Police Service (SAPS). The purpose of this review was to describe the occupational hazards, injuries and diseases affecting police officers worldwide, in order to benchmark policy implications for the SAPS.

**Methods:**

We conducted a systematic review of studies using Google Scholar, PubMed and Scopus.

**Results:**

A total of 36 studies were included in this review. Six revealed that police officers’ exposure to accident hazards may lead to acute or chronic injuries such as sprains, fractures or even fatalities. These hazards may occur during driving, patrol or riot control. There were two studies, which confirmed physical hazards such as noise induced hearing loss (NIHL), due to exposure to high levels of noise. Three studies on chemical hazards revealed that exposure to high concentrations of carbon dioxide and general air pollution was associated with cancer, while physical exposure to other chemical substances was linked to dermatitis. Four studies on biological hazards demonstrated potential exposure to blood borne diseases from needle stick injuries (NSIs) or cuts from contaminated objects. One study on ergonomic hazards showed that musculoskeletal disorders can result from driving long distances and lifting heavy objects. There were 15 studies that indicated psychological hazards such as post-traumatic stress disorder (PTSD) as well as stress. Moreover, four studies were conducted on organizational hazards including burnout, negative workplace exposure and other factors.

**Conclusions:**

This review outlined the global impact of occupational hazards, injuries and diseases in the police force. It served as a benchmark for understanding the policy implications for South Africa, where there is paucity of studies on occupational health and safety.

## Introduction

Exposure to occupational hazards can lead to work-related injuries or diseases [1]. The International Labour Organization (ILO) revealed that every 15 s, 153 employees experience work-related accidents, worldwide. Furthermore, every year 6300 deaths occur due to occupational accidents and 2.3 million deaths occur due to occupational diseases. Apart from death, occupational hazards can lead to prolonged absence from work if not prevented [2]. Annually, a global estimate of 4 % of the Gross Domestic Product (GDP) is lost as a result of occupational injuries and diseases [3]. Since 2001, there has been an increase in the number of reported cases worldwide [4]. In addition, the increase in exposure to air pollution, toxic chemicals and strenuous work activities have negative implications on the health and wellbeing of employees [5]. These observed impacts pose serious health and safety problems. Hence, there is need for inculcating a safety culture in the workplace [6].

The occupational health and safety (OHS) of police officers including in Sub-Saharan Africa is a major public health concern [7]. The ILO expects countries to improve OHS management by creating universal awareness on OHS. However, most developing countries do not have sufficient evidence on the rates or economic costs associated with OHS. Few studies have provided fragmented evidence that requires enormous assumptions to permit simplification and extrapolation [8]. There are limitations with regard to research in the area of OHS, which affects policy development and program management in South Africa. Studies conducted from other countries such as Romania revealed that time spent in attending to occupational injuries and diseases could have been used to combat crime [9]. This has far-reaching consequences in terms of managing crime, whereas in the interest of public health and safety, there is a need to protect police officers. Thus, it is essential to draw lessons from other countries.

In South Africa, the OHS of police officers needs to be seriously considered because the South African Police Service (SAPS) plays a critical role in crime prevention [10]. There is a need to understand how occupational hazards affect the workforce, in order to provide well-planned OHS interventions and a conducive working environment. It can also help address public health concerns and enhance the effectiveness of police officers while on duty [11]. Hence, the objective of this review was to describe the occupational hazards, injuries and diseases affecting police officers worldwide, in order to benchmark the policy implications for the SAPS.

### OHS in the south African police force

In the SAPS, the incidence of injuries sustained while on duty among police officers was 4.7, 6.8 and 4.3% for 2013/14, 2014/15 and 2015/16, respectively [12, 13, 14]. For the same reporting periods, the fatalities were 3.5, 1.2 and 1%, respectively. Although there was a decline in the fatalities, there has not been a significant change to injuries. These findings were observed from the 138,595 police officers working in the SAPS. From the available statistics, the injuries encountered by police officers were not categorized according to the ILO hazard classification [15]. The lack of hazard classification limits the possibility of understanding the common causes of injuries that affect police officers while on duty [16]. Thus, it is essential to have a good OHS plan whereby the employees, employers and the government participate [17].

Currently in South Africa, OHS is well emphasized in other work environments such as the mining and engineering field [6]. Such efforts have proven to be effective in reducing the effects of occupational hazards, because the employer and employees have embraced a safety culture. In Australia, there is emphasis on stress management, training and support for police officers [18]. Thus, it is essential to learn from other countries, how OHS in the police force can be improved. These lessons can help structure policies for effective OHS among police officers in the SAPS.

**Fig. 1:**
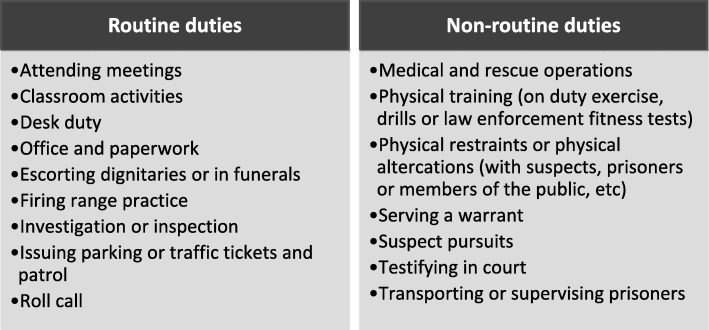
Types of duties related to sudden cardiac death summary of police officers. Source: Varvarigou et al. (2014) [20]

Due to the need for public safety, the police force has a mandate to protect citizens and enforce law [19]. Such duties require both physical and psychological wellness, given that each role is associated with specific hazards. The roles and responsibilities of police officers vary; they include attending the crime scene, patrol, firing range practice or testifying in court. A study from the USA revealed the types of duties related to sudden cardiac death summary of police officers, as shown in Fig. 1 [20]. These duties are categorized as either routine or non-routine. The routine duties are generally conducted as part of daily tasks, while the non-routine duties are those conducted during unforeseen circumstances.

### Types of duties related to sudden cardiac death summary of police officers

**Fig. 2:**
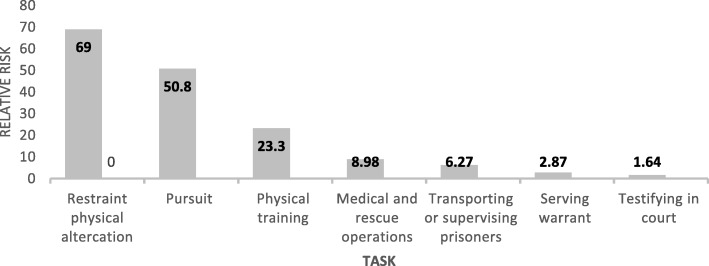
The relative risk of sudden cardiac death based on duties performed by police officers. Source: Varvarigou et al. (2014) [20]

The risk of injury or death depends on the duties performed by police officers [21]. A study conducted in the United States of America (USA), revealed that the relative risk of sudden cardiac arrest among police officers whilst restraining offenders was 69 and 1.64 for testifying in court as shown in Fig. 2 [20]. Non-routine duties (for examples see Fig. 1) increase the risk of exposure of assault or shooting, which may lead to fatality of police officers. The outcomes of hazards related to certain duties can seriously compromise the health and safety of police officers on a short or long-term basis. Thus, it is essential to have an in-depth knowledge of occupational hazards and their effects, in order to take remedial action.

### OHS among police officers

Employers have the responsibility to offer OHS to their employees [22]. OHS is not only a human right; it also provides health, social and economic benefits to both parties. OHS promotion enhances leadership and management in organizations [23]. It results in the productivity and innovation of employees [24]. In addition, it increases the morale, job satisfaction and efficiency in the workplace [25]. It also helps to assess the return on investments and resource allocation [8]. Evidence from other countries such as Australia, have shown positive outcomes on cost-effectiveness of OHS programs for health, productivity and economy [26]. Hence, the development of OHS is often an investment rather than an economic burden [1].

Well-organized OHS programs contribute towards the prevention of injuries and management of diseases [19]. Reporting the burden of occupational injuries or diseases helps with effective implementation of OHS programs [24]. Effective management of OHS can reduce the ripple effects of work-related hazards on the burden of disease in developing and middle-income countries [27]. Without effective OHS programs, it may not be possible to achieve overall wellness. Furthermore, given that a large number of police officers work for the SAPS, it is important to identify the occupational hazards, injuries or diseases which affect them.

Identifying the occupational hazards and its effects in the police force, can help provide management strategies for improving the health and safety of police officers, so that protection of citizens against crime is not compromised. Research has shown that healthy and safe work environments facilitate good working relationships [28]. In addition, good OHS management can enhance efforts in fighting escalating crime rates [25]. The long-term effects include a reduction of associated costs, as well as community satisfaction of the services offered by police officers.

### Public health and cost implications of work-related hazards

OHS injuries and diseases affect the physical, social and psychosocial wellbeing of police officers, which has various public health implications [29]. Some studies indicate that police officers are at risk of dying at a younger age than the general population. In 2011 and 2012, the fatality rate of patrol officers in the USA was 15 to 16 per 100,000 full time workers, respectively [20]. Another study demonstrated that life expectancy among Romanian police officers was 62 years compared to national average of 75 years, as a result of exposure to various workplace hazards in the shorter and longer terms [9].

**Fig. 3:**
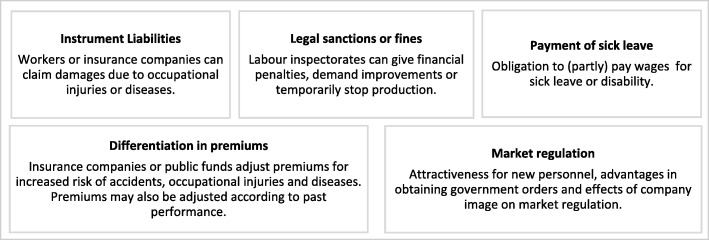
The cost implications of occupational injuries and diseases. Source: Van Ooteghem (2006) [32]

The cost implications of poor OHS management for police officers are associated with reduced wages or unpredicted healthcare expenses [30]. The increase in medical expenses and loss of earnings, leads to loss of goods necessary for livelihoods [8]. The families of the affected police officers also suffer financially and emotionally [31]. In general, employers incur direct OHS costs mainly from insurance premiums and legal settlements [30]. The direct costs often relate to disruption of services from increased sick leave or reduced productivity [32]. The indirect costs relate to training, compensation and loss of working days. Furthermore, the Government may compensate medical bills that employers cannot reimburse [8]. For instance, when employees acquire work-related diseases that cannot be linked to work. The cost implications of OHS related hazards is summarized in Fig. 3 below.

## Materials and methods

### Search strategy

Literature searches were conducted to identify papers on occupational hazards, injuries and diseases experienced by police officers worldwide, in order to benchmark the policy implications for the SAPS. Searches were done using Google Scholar, PubMed and Scopus on articles published from 1987 to 2017. The following key words were used; police and occupational diseases, occupational hazards, occupational health and safety, occupational injuries, stress, work injury.

### Inclusion criteria and exclusion criteria

**Fig. 4:**
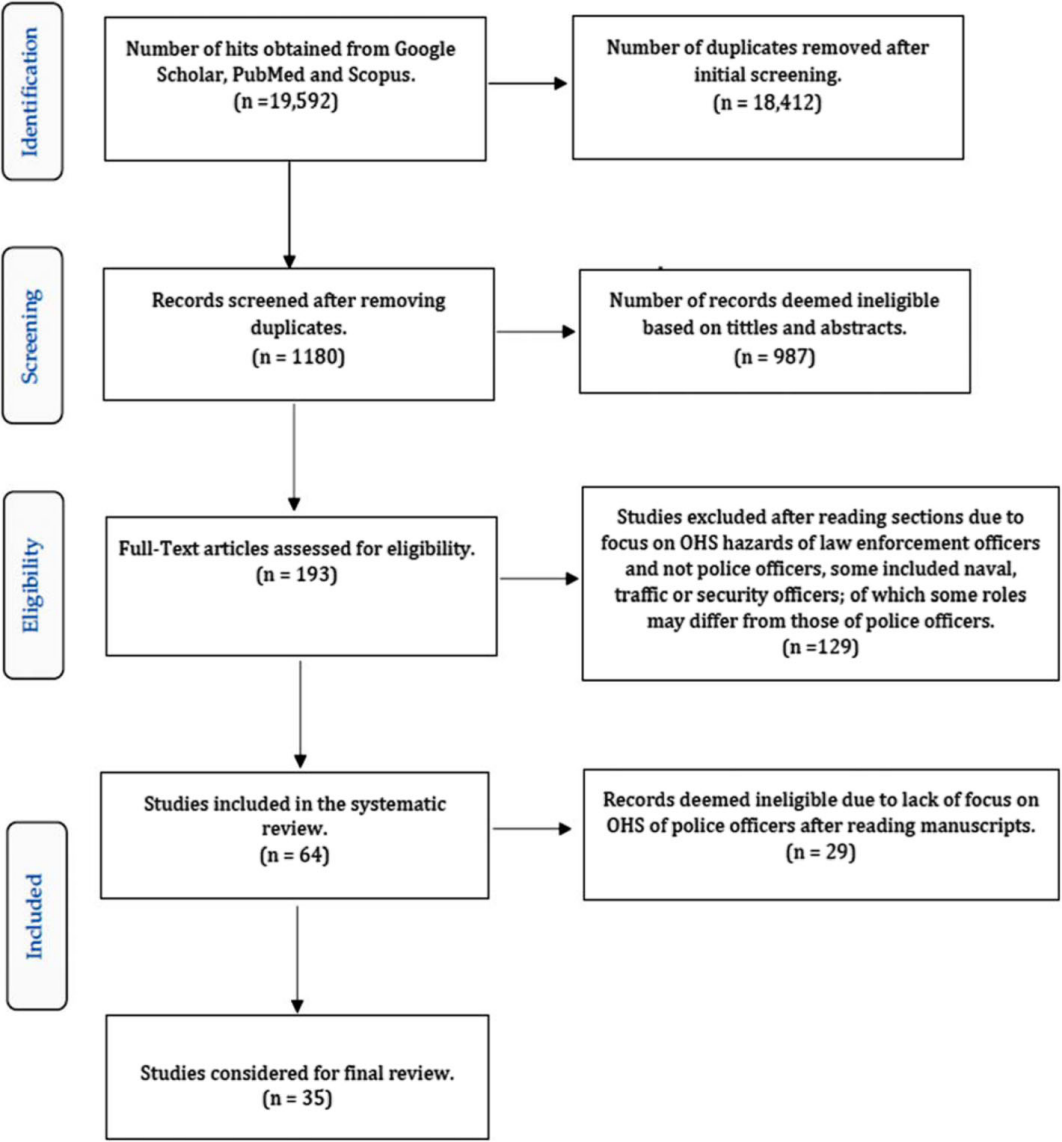
PRISMA flow diagram

Studies deemed eligible for this review were those published in English in internationally peer-reviewed journals. The eligibility of studies was based on the relevance to hazards, injuries and diseases affecting police officers in their line of duty. Where there was doubt of study findings, the three authors had a discussion and agreed on what could be excluded. Figure 4 provides an overview of the inclusion and exclusion criteria.

From the searched databases, we identified 19,592 records. Of these, 18,412 duplicates were removed. The remaining 1180 records were screened. A total of 987 records were excluded because they were deemed ineligible based on abstracts and titles. Only 193 full-text articles were assessed for eligibility. We excluded 129 studies, because although they were on occupational hazards, injuries and diseases of law enforcement officers; they were not specific to police officers. Some of the excluded studies included the naval, traffic or security officers whose work roles may differ from those of police officers. From the 64 studies considered for the systematic review, 29 were excluded, because they lacked sufficient data on occupational hazards, injuries and diseases specific to police officers. Thirty-five studies were considered for the final review. The included studies covered qualitative and quantitative synthesis of various occupational hazards, injuries and diseases among police officers globally.

## Results

**Fig. 5:**
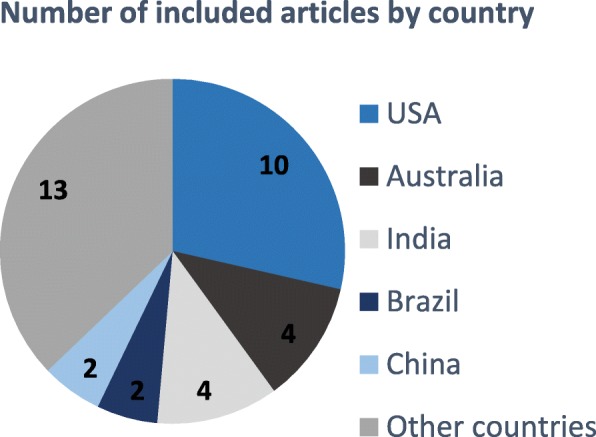
Number of articles included in the study by country

The ILO categorizes hazards that affect police officers as accident, physical, chemical, biological, ergonomic, psychological and organizational [33]. From the 36 studies in this review, we identified the occupational hazards, injuries and diseases affecting police officers in the workplace. The papers included in this review are highlighted in Fig. 5 and Table 1.

**Table 1:** Summary of assessed studies (1987 and 2017) on occupational hazards and related diseases among police officers worldwide

Author and Year	Objectives	Study area	Methods	ILO Category of occupational hazards	Outcomes
Ellis (1993) [36]	To determine the dangers of domestic conflicts and identify factors increasing the risks of injury to police officers.	Canada	Survey	Accident	Factors that led to injuries were attempting to arrest suspects or answering a domestic disturbance call alone. The police were exposed to verbal or physical abuse, intoxication and physical injury from the suspects.
Larsen et al. (2016) [37]	To investigate the injuries sustained by the Australian specialist police division.	Australia	Cohort study	Accident	Half of the reported injuries occurred during operational policing tasks, while more than 30% of injuries occurred during training activities. Strain or sprain and upper body injuries were almost five times more common than lower-body or torso injuries.
Ferguson et al. (2011) [38]	To examine the prevalence of work-related injuries and occupational violence experienced by police officers from 2000 to 2008.	Australia	Case-control study	Accident	The police officers were among the top three highest claiming occupations for occupational injuries and deaths when compared with security officers. Stress accounted for 87.3% of injuries among police officers.
Thejus et al. (2014) [39]	To identify injuries, associated factors and effects on the health of police officers.	India	Cross-sectional study	Accident	Police officers were susceptible to accident hazards. These hazards led to injuries and accidents. Lacerations were on the increase when compared to fractures.
Brand I (1996) [40]	To compare assault and accident rates, in order to understand accidental injuries and deaths among police.	USA	Case reports and series	Accident	Most injuries were as a result of accidents based on 2073 police injury incident reports. Accidents led to increased number of injuries, medical treatment and days of work lost.
James, S.M. (2015) [41]	To examine the effect of distraction caused by the use of text while police officers are driving.	USA	Controlled laboratory experiments	Accident	Lane deviations resulting from distracted driving increased the risk of collision. Motor vehicle accidents were common among police officers, whose role required them to drive while at work.
Lesage et al. (2009) [42]	To evaluate the association between police employment and noised induced hearing loss (NIHL).	France	Cross-sectional study	Physical	Police officers were 1.4 times at risk of hearing loss compared to other civil servants. There was an observed difference between motorcycle operators and civil servants.
Win et al. (2015) [43]	To estimate the prevalence of NIHL in the police force.	Brunei	Cross-sectional study	Physical	Of the reported NIHL cases, 74.8% were police officers. A strong association between NIHL and male sex, increased age as well as longer duration of service was observed. Overall, mild NIHL was 93%, moderate NIHL was 3.5% and severe NIHL was also 3.5%.
Gu et al. (2011) [44]	To examine the incidence of cancer in white-male police officers.	USA	Cohort study	Chemical	Between 1976 and 2006, the risk of brain cancer was slightly higher among police officers than in the general population. Police officers who worked for more than 33 years had an increased risk of Hodgkin’s lymphoma.
Chen et al. (2015) [34]	To evaluate exposure of particulate matter (PM) concentrations in Sinying police station near a highway.	Taiwan	Case-control study	Chemical	There was a higher carbon dioxide concentration on the selected police station due to emission sources and indoor air pollution than the reference station. Police officers working along the busy roadways were exposed to high concentration of air pollution.
Treudler et al. (1999) [46]	To evaluate the effect of 2-chloracetophenone (CN) on occupational contact dermatitis.	Germany	Case reports and series	Chemical	Localized dermatitis was observed on contact site of three police officers who experienced the accidental escape of CN, from professional tear gas canisters. Patch tests revealed allergic reactions, which led to extensive health problems.
Mittal et al. (2016) [47]	To evaluate prevalence of needle stick injuries (NSIs) among police officers.	Mexico	Survey	Biological	Police officers reported incidence of NSIs. Contributing factors were exposure to syringes with drugs and breaking of used needles. Police officers were predisposed to various blood borne diseases such as the Human Immunodeficiency Virus (HIV).
Lorentz et al. (2000) [48]	To assess the risk of NSIs and determine predictors of injuries and reporting rates among officers.	USA	Survey	Biological	Of the respondents, 29.7% had at least one NSI while 27.7% of the group had two or more. Risk factors included pat-down searches, working evening shifts, male gender, patrol duties and less years of experience. Only 39.2% of participants sought medical attention following a NSI.
Merchant et al. (2008) [49]	To estimate incidence of emergency department visits due to blood or body fluid exposures on utilization of HIV post-exposure prophylaxis (PEP) by police officers.	USA	Case-control study	Biological	The average incidence rate of emergency department visits due to blood or body fluid exposures was at 4.41 per 1000 officers. Of the officers, 15% sustained percutaneous injuries or blood-to-mucous membrane exposures.
Averhoff et al. (2002) [50]	To determine the frequency and type of occupational exposures and risk of hepatitis B virus infection experienced by public safety workers.	USA	Survey	Biological	Of the participants, 6.8% reported at least one occupational exposure within six months. Human bites were 3.5%, cuts from a contaminated object were 2.8% and NSIs were 1.0%, while mucous membrane exposures to blood were 0.9%.
Cho et al. (2014) [51]	To examine the status and prevention of musculoskeletal disorders in Korean police officers.	Republic of Korea	Case reports and series	Egornomic	The shoulder disorders were 4.87 times higher in police lieutenants than corporals. For police officers with chronic diseases, the risk was 1.78 times higher than in those without chronic diseases.
Ma et al. (2015) [53]	To examine the association between shift work and police work-related stress.	USA	Survey	Psychological	There was a higher risk of PTSD among police officers. The common factors were increased exposure to life-threatening or traumatic events, negative thoughts about work, lack of social support and shift work.
Maia et al. (2007) [54]	To determine prevalence of post-traumatic stress symptoms and compare morbidity among police officers.	Brazil	Survey	Psychological	The prevalence rates of “full PTSD” and “partial PTSD” were 8.9 and 16%, respectively. Compared with the “no PTSD” group, police officers with “full PTSD” had additional medical consultations during the previous year, reported poor physical health and more lifetime suicidal ideation.
Maia et al. (2008) [71]	To measure the serum lipid composition on police officers with and without PTSD who are regularly exposed to potentially traumatic situations.	Brazil	Cross-sectional study and Medical evaluation	Psychological	Police officers with PTSD presented an increased serum total cholesterol and low-density lipoprotein levels than those without PTSD. Abnormal serum lipid profile and higher body mass index (BMI) suggests that PTSD increases the risk of developing metabolic syndrome.
Houdmont et al. (2012) [11]	To study the association between exposures and perceived work-related stress on police officers.	United Kingdom (UK)	Survey	Psychological	There was evidence of highly stressful work as reported by the police officers. Such stresses were linked to the nature of duties performed by police officers.
Garbarino et al. (2015) [56]	To evaluate the association between occupational stress and metabolic syndrome (MetS) in a rapid response police unit.	Italy	Medical evaluation and Survey	Psychological	Majority of police officers reported high stress levels. There was a higher risk of developing MetS and hypertriglyceridemia among police officers. Work-related stress proved to induce MetS among police officers.
Hartley et al. (2011) [58]	To examine the association between stress and MetS with its individual components among police officers.	USA	Survey	Psychological	The stress with MetS constituents was elevated among female, when compared to male police officers. Perceived stress, administrative or organizational pressure and lack of support was associated with the MetS among female police officers.
Janczura et al. (2015) [59]	To examine whether higher levels of stress might be related to MetS and plaque presence, and whether MetS might affect pulmonary function.	Poland	Medical evaluation	Psychological	Job-specific stress increased with the prevalence of MetS and affected coronary plaque presence among police officers. Leisure-time physical activity proved to reduce the risk of developing MetS, while MetS subjects had worse parameters for pulmonary function.
Malach-Pines (2006) [57]	To map stressors that effect Israeli border police, assess its outcomes and suggest ways to reduce burnout and stress.	Israel	Survey	Psychological	The police officers reported lack of resources, low salary and overload as stressors. Those who reported traumatic experience such as a terrorist attack were 74%, while 52% reported high levels of stress. The burnout level among police officers was 4.15 compared to the national burnout level of 2.8.
Berg et al. (2006) [35]	To explore physical and mental health among Norwegian police and associations to job stress.	Norway	Survey	Psychological	Frequent job pressure and lack of support was linked to physical and mental health problems. Anxiety symptoms in females were 4.2 and 3.7 among males. Depressive symptoms were 3.1 among males and 2.4 among females.
Ramakrishnan et al. (2013) [69]	To assess the prevalence of cardiovascular risk factors including stress among police officers.	India	Cross-sectional study and Medical evaluation	Psychological	There was an increase in cardiovascular risk factors such as stress, diabetes and hypertension among police officers. Moderate stress related to work and life problems was 70 and 4%, respectively. The prevalence from the 23% of known diabetic patients, was 33.6%, while from the 16.8% unknown hypertensive patients, it was 30.5%.
Ramey et al. (2011) [67]	To explore risk factors for cardiovascular disease such as stress among Midwestern police officers.	USA	Survey	Psychological	There was a significant relationship between stress and cardiovascular disease and vital exhaustion. A change in unit increased the odds of cardiovascular diseases by 20% on perceived stress and 31% on vital exhaustion among police officers.
Walvekar et al. (2015) [60]	To examine the association between perceived stress, Serum Cortisol and explore stress leading to metabolic syndrome.	India	Survey and Medical evaluation	Psychological	There was a positive link between Serum Cortisol and perceived stress scale, blood glucose and heamoglobin A1c. Among police constables, the biochemical parameters were elevated than the general population. Of the police officers, 38% had cardio metabolic syndrome.
Ganesh et al. (2014) [70]	To assess the prevalence and risk factors of hypertension such as stress among male police personnel.	India	Cross-sectional study	Psychological	Perceived stress was 51%, obesity 54.4%, smoking 21.6%, alcohol use 50.3% and diabetes 10.1%. Higher perceived stress, alcohol use and diabetes were significantly linked to hypertension.
Chen et al. (2015) [74]	To study links between psychological distress and dyslipidemia among police officers.	China	Survey and Symptom checklist	Psychological	In the two and half years of the study, 60.5% of participants developed dyslipidemia. These findings confirmed the relationship between psychological distress and dyslipidemia
Queirós et al. (2013) [75]	To investigate relationship between burnout and aggressivity among police officers.	Portugal	Cross-sectional study	Psychological	There was a relationship between burnout and aggressivity. Feelings of low personal achievement and high depersonalization contributed to anger. While emotional exhaustion was associated with verbal aggression.
Gu et al. (2012) [63]	To investigate the relationship between long work hours and adiposity measures in police officers.	USA	Case reports and series	Organizational	There was an association between long work hours, increased waist circumference and body mass index. The common factors were working midnight shifts, poor eating habits and increased work stress, which could lead to coronary heart diseases.
Kuehl et al. (2016**)** [68]	To determine changes in blood pressure and associations with the overall sleep quality and fatigue among police officers.	Australia	Cross-sectional study	Organizational	From the participants, 69% reported poor sleep quality and 51% reported severe fatigue. There was an increase of systolic blood pressure across the shifts among female participants, while there were no changes in blood pressure among the male participants.
Tuckey et al. (2010) [65]	To examine mental and cardiovascular health problems related to negative workplace environment exposure.	Australia	Survey	Organizational	Evidence of negative workplace behaviour and cases of bullying affected the cardiovascular outcomes and psychological wellbeing of employees. The negative behaviour led to physical disease and psychological illness.
Yu et al. (2016) [66]	To explore links between occupational stressors and type 2 diabetes mellitus among police officers.	China	Cohort study	Organizational	Stressors increased with the number of years in police work and resulted in new onset diabetes (NOD) among participants. There was a relationship between occupational stressors and abnormal glucose metabolism. In 2008, 2009, 2010 and 2011 the incidence rate of NOD were 0.58, 0.98, 0.52 and 1.01%, respectively.

### Accident hazards

Exposure to accident hazards such as vehicle crashes, falls and assaults may lead to acute or chronic injuries such as sprains, fractures or even fatalities among police officers [33]. Our findings revealed that accident hazards may occur during driving, patrol or riot control [34]. Police officers can accidentally injure themselves or suffer from injuries sustained during restraining suspects [35]. Six studies confirmed that police officers are prone to accident related hazards. A study among 646 patrol officers in Canada, showed that some accidents occurred as a result of attempts to make arrests or lack of preparation for the task at hand [36].

A study conducted in the Australian specialist police division, showed that half of work-related injuries occurred during operational policing tasks, while more than 30% of those were related to training activities [37]. The injuries led to a loss of 1107 shifts and an estimated monetary loss of $487,159, over four years. Another study conducted across Australia, revealed more claims for occupational injuries, violence and deaths among police officers [38]. There were higher rates of fatal injury at work among police (7.3 per 100,000) than security officers (3.8 per 100,000). Vehicle accidents were the most common causes of deaths among police officers.

Furthermore, a cross-sectional study among 900 policemen in India revealed that 20% of police officers reported injuries sustained while attending to accidents [39]. The causes of injuries were mainly encounters (52.7%) and accidents (35.5%). Lacerations (43.2%) and fractures (36.7%) were the commonly reported injuries. A study among 2073 officers from the Midwestern municipal police department in the USA confirmed that most reported injuries resulted from accidents and have led to many days of work loss [40]. Another study from the USA among 80 experienced police patrol officers, revealed that distraction during driving increased the chances of lane deviation [41]. Such distractions increased the susceptibility of police officers to motor vehicle collisions.

### Physical hazards

Physical hazards in the police force can occur due to high levels of noise, related to the firing range or emergency horn [33]. Two studies provided details regarding noise induced hearing loss (NIHL) in the police force. In a study conducted in France among 1692 police officers, it was indicated that police officers were almost two times more likely to have hearing loss, compared to ordinary civil servants [42]. Motorcycle operators were among the police officers most affected by NIHL. From a study conducted on 543 police officers working for Royal Brunei Police Force, only 64.4% used hearing protective devices during shooting practice [43]. The 74.8% of cases with NIHL were police officers. In total, the incidence of mild, moderate and severe NIHL was 93, 3.5 and 3.5%, respectively. A higher prevalence of 37.7% was observed among males than the 23.9% on females. From this study, there was a strong association of NIHL with duration of service, age, rank, hypertension and diabetes mellitus.

### Chemical hazards

The ILO indicates that exposure to lead during traffic control, finger-printing work or working on the firing range can be harmful [33]. Furthermore, exposure to excessive carbon monoxide levels when directing traffic can affect the health of police officers. Findings from three studies demonstrated serious health effects related to chemical hazards. A study from the USA among a cohort of 2234 police officers in Buffalo revealed that over a period of 31 years, 18.2% of the participants developed cancer associated with vehicle emissions near the highway [44]. Compared to the general population whose standardized incidence ratio was 1.61, the SIR among police officers with 30 or more years of service increased significantly to 2.92.

Moreover, a study conducted in Taiwan among police officers working at Sinying police station showed a higher carbon dioxide concentration due to emission sources and indoor air pollution, compared to a reference station [34]. Police officers working on the busy highway were exposed to high concentrations of air pollution. Such exposures are often associated with severe health effects including cardiovascular or respiratory diseases [45].

Furthermore, findings from a study conducted among three German police officers confirmed that constant use of 2-Chloracetophenone (CN), a tear gas, for self-defense can result in respiratory, eye and skin problems [46]. From the three police officers who experienced accidental escape of CN from their professional tear gas canisters, all presented with localized dermatitis on contact site with the CN. A patch test dissolved in acetone revealed an allergic and an irrigative reaction on the affected police officers. Over the years, there has been an observed increase in the number of occupational contact dermatitis cases due to CN among police officers.

### Biological hazards

Biological hazards involve the risk of contracting contagious diseases such as HIV, infectious hepatitis or rabies. The causes often include needle stick injuries (NSIs), close contact with infected people or human bites [33]. Four studies revealed that biological hazards affect police officers. Results from a survey conducted in Mexico in the Tijuana police force showed that 16.7% of police officers reported NSIs while at work [47]. The contributory factors to NSIs were direct contact with syringes that contain drugs and broken used needles. These exposures could result in contamination from blood-borne infections. A study in the San Diego Police Department confirmed that NSIs occurred frequently among police officers, which exposed them to HIV, hepatitis B and C [48]. From the participants, 29.7% reported at least one NSI while 27.7% reported two or more. The risk factors included working evening shifts, patrol duties and less years of work experience.

Another study from Rhode Island in the USA, revealed that 4.41 per 1000 officers undertook emergency department visits due to blood or body fluid exposures [49]. The findings of this study revealed an increased number of post-exposure prophylaxis (PEP) for HIV offered to police officers. The number of police officers who required PEP due to blood-to-mucous membrane exposures was three times higher, when compared to other body fluid exposures. Moreover, findings from a survey conducted in the USA among 2910 public safety workers including police officers, demonstrated that 6.8% of participants reported at least one occupational exposure in a period of six months [50]. Incidence of human bites was 3.5% and for cuts with contaminated objects was 2.8%. Exposure to NSIs were 1.0%, while exposure to mucous membrane containing blood was 0.9%.

### Egornomic hazards

**Fig. 6:**
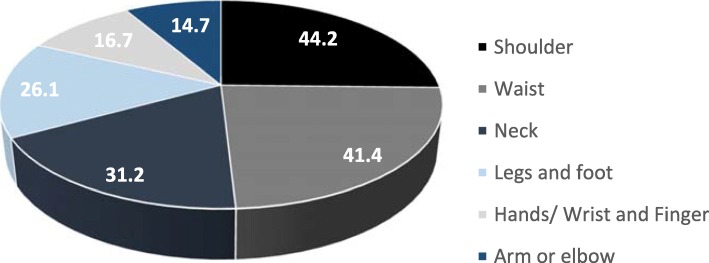
The types of pain on body parts as reported by police officers. Source: Cho et al. (2014) [55]

Prolonged driving can lead to musculoskeletal disorders such as low back pain [33]. One study from this review, focused on ergonomic hazards that led to musculoskeletal disorders among police officers. A study among 353 police officers who made a previous visit to the National Police Hospital in Korea, revealed that police officers with chronic diseases had higher risks of developing musculoskeletal disorders than their counterparts without a history of chronic diseases [51]. The upper part of the body was reported to be more prone to musculoskeletal pain. Figure 6 demonstrates that shoulder pains were reported more often (44.2%) than elbow or arm pains (14.2%).

### Psychological hazards

Emotional trauma is a common psychological hazard which can affect police officers, due to witnessing traumatic events, horrific incidents and psychological stressors [33]. Other traumatic events include physical injury, assault or hostage situation [52]. The highest number of studies obtained for this review reported on psychological hazards. A study in the USA among 365 police officers, revealed a higher risk of post-traumatic stress disorder (PTSD) among police officers, due to exposure to life-threatening events [53]. There were concerns about work pressure, physical and psychological dangers associated with police work. Furthermore, in a Brazilian study among 157 police officers from the elite unit, a prevalence of “full PTSD” was linked to considerable morbidity [54]. The prevalence rates of “full PTSD” and “partial PTSD” were 8.9 and 16%, respectively. Compared with the “no PTSD” group, police officers with “full PTSD” reported poor physical health and indicated exposure to lifetime suicidal ideation.

Moreover, police officers are classified under emergency personnel, hence they are prone to stress [55]. Stress affects the psychological, social and emotional life of police officers. A study conducted in the United Kingdom (UK) among London police officers revealed that 46% of police officers reported work to be extremely stressful [11]. While in Italy, a longitudinal study conducted among 290 police officers demonstrated high stress levels [56]. A study among 497 Israeli border police, revealed that lack of resources, low salary and work overload were common stressors in the workplace [57]. Similarly, a survey on 3272 Norwegian police officers demonstrated that frequent job pressure and lack of support was associated with physical and mental health problems [44]. Various studies have shown that police officers with psychological problems such as PTSD or stress presented with increased total cholesterol levels [58, 59]. Psychological problems may lead to other health risks including diabetes and hypertension.

The negative physical health effects resulting from psychological hazards can vary from one police officer, to another. In India, a study conducted among 108 male police constables from the Bijapur demonstrated a positive correlation between serum cortisol and perceived stress scale [60] associated with psychological problems. Police constables had elevated biochemical parameters than the general population. Of the police officers studied, 38% suffered from cardio metabolic syndrome. Two studies confirmed that stress among police officers increased the prevalence of stress and metabolic syndrome (MetS) [58, 59]. In addition, a survey conducted among 5867 police officers in Tianjin, China, has shown a positive link between psychological distress and dyslipidemia [43]. Among 3300 participants without dyslipidemia at baseline, 60.5% developed dyslipidemia two years later.

Another negative effect of psychological problems among police officers is burnout [61]. A study from 274 male police officers from Portuguese Police of Public Security in Porto and Lisbon, revealed that burnout also affected the emotional stability of police officers [62]. Burnout accounted for 13%, while aggression was 22% among police officers. Police officers with feelings of low personal accomplishment and high depersonalization had anger and emotional exhaustion, which accounted for 4 % of verbal aggression.

### Organizational hazards

Organizational hazards such as shiftwork, poor relations with superiors in a hierarchical system, can have negative effects on the health of police officers [33]. Four studies in this review demonstrated the effect of organizational hazards among police officers. In the USA, among 408 officers from the Buffalo, work shifts and prolonged sleep deprivation were associated with weight gain [63]. In Australia, a study among 206 police officers from the New South Wales Police Force, revealed that shift work increased blood pressure among female participants [64]. From the participants, 69% reported poor sleep quality and 51% reported severe fatigue.

Another study in Australia conducted on 251 police officers, revealed a significant relationship between poor cardiovascular health, mental health problems and negative behaviour in the workplace [65]. A study on occupational stressors conducted in Tianjin, China among 6559 police officers reported that 3.1% of the participants developed new-onset diabetes (NOD) within four years [66]. In 2008, 2009, 2010 and 2011, the NOD incidence rates were 0.58, 0.98, 0.52 and 1.01%, respectively.

## Discussion

This review provided detailed information regarding the occupational hazards, injuries and diseases that affect police officers while on duty. Most of the studies reviewed were conducted in the USA [40, 41, 44, 48–50, 53, 58, 67, 63], Australia [37, 38, 68, 65], India [39, 69, 60, 70], Brazil [54, 71] and China [34, 66]. We also found studies from Brunei [43], Canada [36], France [42], Germany [46], Israel [61], Italy [56], Korea [51], Mexico [47], Norway [35], Poland [59], Portugal [62], Taiwan [34] and the UK [11]. From these studies, it was clear that occupational hazards can result in various injuries and disease [40]. In the police department, affected police officers are off work for a considerable number of days or months, which leads to loses in working hours. Hence, it is essential for the SAPS to develop guidelines to effectively manage related occupational exposures.

This review has shown that police officers are at a higher risk of accident related hazards, which lead to injury, compared to non-police officers who work for the same department [36–41]. This review also identified the common causes of work related injuries and diseases, as well as how it compromise effectiveness of police officers in discharging their duties [33]. For example, a well experienced police officer who sustains a fracture from a fall, may struggle to pursue criminals in the future. Thus, it is important to promote safe driving and conduct regular in-service training on the prevention of accident hazards [72].

Physical hazards such as increased noise levels from the firing range or emergency horn cannot be completely avoided by police officers; this is because such duties are part of their routine work [64]. However, given the potential of long-term hearing impairment, there is need to introduce and implement noise control measures. The SAPS requires guidelines that will address NIHL, including annual hearing exams for monitoring the hearing of police officers, as well as ear protection when at the firing range. It is also key to address other physical hazards that can occur due to exposure to ambient environmental factors, such as adverse weather conditions. There should be good provision of personal protective clothing and equipment for officers who work in harsh weather conditions.

From our findings, it is evident that exposure to chemical hazards can lead to cancer, which is a major concern among police officers [42]. Furthermore, exposure to CN has been shown to result in occupational dermatitis [54]. Therefore, it is important for police officers to be screened for cancer annually. In addition, the SAPS need to educate police officers about skincare and provide sunscreen cream. Such efforts can help prevent, identify and manage the risk of cancer in the police force.

Biological hazards can affect police officers due to contact with human beings, as part of routine work. The reports of NSIs while on duty, demonstrated how important it is to manage biological hazards in the police force [50]. There should be guidance on how police officers can deal with such hazards. It is often difficult to prove the association between biological hazards and medical conditions contracted while on duty [47]. Hence, the policies should indicate how to report NSIs and other work-related incidents when they arise. Furthermore, police officers should be encouraged to report incidents early, in order to obtain PEP.

Ergonomic hazards arising from prolonged driving or lifting of heavy items in the police force requires careful consideration. Moreover, the impact of ergonomic hazards on the musculoskeletal system of the affected police officers cannot be overlooked [73]. The SAPS need to alleviate frequent job pressures by hiring more police officers where there are shortages [35]. When the police officers work in teams, they can take turns to drive, to reduce the effect of fatigue. Training regarding correct lifting techniques should be provided, in order to ensure compliance.

The findings on psychological hazards such as PTSD and stress can affect the mental and physical health of police officers [11, 35, 56–60, 67, 69, 71, 74, 75]. It is evident that police officers who suffer from stress, are more likely to suffer emotional exhaustion [62]. Police work is structured to show less emotion hence, it is difficult for them to seek medical attention when they experience psychological problems [76]. There is a need for effective post traumatic counselling and stress management in the SAPS [62]. There should be a consideration for daily routine work and operational duties, when assessing the health of police officers [35]. Moreover, there should be education provided on steps to be taken during traumatic situations in the workplace, to boost the morale of police officers.

Organizational hazards can affect the physical and mental health of police officers. Poor mental health is a serious social and public health burden that requires management [77]. If not addressed, mental health problems in the police force can lead to a serious public health crisis [78]. Therefore, it is important to develop and reinforce a policy that creates enabling environments, to reduce poor health outcomes, which could result from organizational hazards.

From the studies in this review, we identified different hazards which police officers are exposed to. We also classified them according to ILO classification of hazards [33]. Most of the identified studies reported on occupational hazards, injuries and diseases that are relevant to the South African context as an upper middle-income country, thus creating a good basis for benchmarking OHS policies. The review also highlights the need for improved knowledge, control measures, management and stakeholder participation [20]. Based on the findings of this study, we recommend that the SAPS develop an overarching OHS policy, to address each hazard identified in this study. Where there are existing policies, it is key to reinforce those through communication and training of the police officers.

Furthermore, the SAPS should utilize evidence-based solutions to manage occupational hazards. There is a need for detailed data with emphasis on the classification of occupational injuries and diseases according to the ILO categories. Moreover, the SAPS can draw lessons from other countries on strategies to improve the OHS. There is a good example on strategies to monitor injuries on duty of police officers from Australia that can help serve as a guide. Moreover, when managing OHS, there is a need for changing the safety culture, in order to have successful OHS programs in the workplace. Safety culture comprises of attitudes, norms, beliefs and practices which minimize exposure of individuals to hazardous conditions related to work [79]. For example, in workplaces where safety culture is part of the organizational culture, it becomes easy for employees to report potential work hazards, without any fear of being victimized [80].

It is imperative to utilize a safety model that is cost effective and user friendly, in order to promote the health and safety of police officers [81]. Based on our findings, the SAPS requires commitment to undertake further research studies on OHS and implement recommendations, in order to manage occupational hazards, injuries and diseases which affect police officers.

## Limitations

The initial aim of the study was to target occupational hazards, injuries and diseases that affect the South African police officers. However, despite the comprehensive search, there was limited literature on the subject for the South African setting, even from the Sub-Saharan Africa. For South Africa, the majority of OHS studies found were in the mining sector and not in the police force. Hence, we reviewed global literature, in order to understand the policy implications for the SAPS.

## Conclusion

This review showed that occupational hazards, injuries and diseases can affect the health and safety of police officers. Thus, there is a need for preventative measures such as educating police officers to reduce occupational injuries and fatalities resulting from accident hazards. Similarly, physical hazards like high noise levels, requires police officers to ensure ear protection. Chemical hazards can be related to high concentration of air pollution exposure that can lead to cancer, hence there is need for police rotation. Biological hazards such as NSIs can lead to diseases such as HIV. Therefore, police officers should be encouraged to report when exposed to NSIs. In addition, ergonomic hazards can be prevented by avoiding prolonged driving to reduce musculoskeletal problems. The SAPS seems to have improved the reporting systems on occupational injuries. However, there should be a comprehensive model for management to identify and analyze the factors contributing to the observed increase in OHS cases. Given the scarcity of information on the OHS of police officers in Southern Africa, more studies in the region are needed. Such studies will inform policy as well as management principles and interventions. When a strategy on the OHS of police officers is well developed and implemented, there will be effectiveness on how the SAPS manages crime in South Africa.

## Abbreviations

BMI: Body mass index
CN: 2-Chloracetophenone
GDP: Gross Domestic Product
HIV: Human Immunodeficiency Virus
ILO: International Labour Organization
MetS: stress and metabolic syndrome
NIHL: Noise induced hearing loss
NOD: New-onset diabetes
NSI: Need stick injuries
OHS: Occupational health and safety
PEP: Post-exposure prophylaxis
PTSD: Post-traumatic stress disorder
SAPS: South African Police Service
UK: United Kingdom
UKZN: University of KwaZulu-Natal
USA: United States of America
